# Drawdown flushing in a chain of reservoirs—Effects on grayling populations and implications for sediment management

**DOI:** 10.1002/ece3.4865

**Published:** 2019-01-15

**Authors:** Walter Reckendorfer, Hannes Badura, Claudia Schütz

**Affiliations:** ^1^ VERBUND Hydro Power GmbH Vienna Austria; ^2^ Department of Botany and Biodiversity Research Universität Wien Vienna Austria

**Keywords:** drawdown flushing, European grayling, reservoir management, sediment connectivity, sediment management, suspended solids

## Abstract

We used an information theoretic approach to assess the effects of an ecologically adjusted sediment management scheme on grayling (*Thymallus thymallus* L. 1758) populations. Additionally to reservoir operation, candidate models included a variety of parameters and processes that may influence grayling populations such as flow, temperature, density dependence, and bird predation. Population parameters analyzed included total densities, young of the year numbers, and larval densities. These analyses were supplemented by a characterization of sediments and sedimentation patterns in the reach. Investigations were carried out in six sites affected by flushing and in one control site. A total of thirteen flushing operations have been undertaken within the study period leading to considerable remobilization of fine sediments and gravel. Due to seasonal and hydrological restrictions, not every flood could be used for flushing. These limitations led to an interrupted management throughout the chain of reservoirs as well as to long time intervals between flushing events with possible effects on spawning habitat quality. None of the investigated population parameters was affected by flushing, and thus, the study generally supports the current reservoir management scheme. Our analyses revealed the magnitude and timing of high water events, temperature, and density‐dependent effects, that is, population densities the year before, as the most influential variables for grayling population dynamics in the investigated stretch. The siltation of reservoirs is a significant problem for reservoir storage, flood protection, river deltas, and coastal zones. Its management—which is inevitable to safeguard river deltas and secure flood protection—poses also the challenge to safeguard riverine ecosystems below reservoirs. Based on our experience, we propose a periodic flushing regime in concordance with the hydrograph thereby mimicking the timing, magnitude, frequency, and duration of natural SSC pulses and gravel transport. This flushing regime minimizes adverse downstream environmental impacts and maximizes benefits.

## 1 INTRODUCTION

Globally, more than 25% of sediment flux is trapped in artificial impoundments (Vörösmarty et al., 2003). Although humans are simultaneously increasing the river transport of sediment through soil erosion activities, the net result is a global reduction in sediment flux by about 1.4 BT/year over prehuman loads with subsequent impacts on coastal ecosystems (Syvitski, Vörösmarty, Kettner, & Green, 2005). For the European Alps, Hinderer, Kastowski, Kamelger, Bartolini, and Schlunegger (2013) estimated that only 45% of the sediments mobilized in headwaters are exported out of the Alps, most sediments being trapped in artificial reservoirs. From this global perspective, it becomes clear that releasing sediments from reservoirs is necessary to safeguard and protect river deltas and other coastal systems.

On a local scale, flushing operations are inevitable to preserve reservoir storage, prolong the lifespan of reservoirs, and secure flood protection (deNoyelles & Jakubauskas, 2008; Kondolf et al., 2014). On the other hand, they have been blamed for negative effects on wild freshwater fish populations and yields (Crosa, Castelli, Gentili, & Espa, 2010; Espa, Castelli, Crosa, & Gentili, 2013; Gerster & Rey, 1994; Rathburn & Wohl, 2001).

The adverse ecological consequences of flushing releases are related both to the increase of suspended sediment concentration (SSC) during the removal operation and to the modification of riverine habitats following deposition of the flushed fine material. Studies on the effects of fine sediment, both suspended and deposited, are numerous and have been reviewed by several authors (e.g., Kemp, Sear, Collins, Naden, & Jones, 2011). It is generally acknowledged that the direct impact differs between species—with salmonids being most vulnerable—and varies with SSC and exposure time. Deposition of the flushed fine material may also have a significant impact on fish populations. Generally, an increase in fine sediments was found to decrease hatching success in salmonids through reducing intragravel O_2_ concentrations (Greig, Sear, & Carling, 2005; Jensen, Steel, Fullerton, & Pess, 2009). As grayling (*Thymallus thymallus* L., 1758)—contrary to other salmonids—do not bury their eggs but lodge them at depths of 2 to 3 cm into the substrate (Northcote, 1993), this relationship may not be so pronounced in this species.

An aspect mostly ignored when assessing flushing operations is the delivery of coarse substrate during flushing. Grayling has very specific requirements on spawning substrate, preferring medium‐sized gravel (20 to 50 mm, Mouton et al., 2008). Sediment supply downstream of dams is greatly reduced, often leading to coarsening of the substrate and armoring (review in Bednarek, 2001). During flushing, these processes may be mitigated and ultimately leading to habitat improvement (Kondolf & Matthews, 1991).

Historically, flushing operations have often been undertaken without consideration of ecological impacts. This led partially to high mortality in fish populations in consequence of flushing (e.g., Hesse & Newcomb, 1982). However, the increase in environmental awareness has led to the recognition that the reservoir management includes a responsibility to protect the natural resources that depend on water. As a result, considerable effort has been invested in developing approaches to lessen the damaging effects of flushing operations including guidance values for SSC, seasonal restrictions, and postflushing with clear water.

The aim of the current paper was to assess the effects of an ecologically adjusted reservoir management on *Thymallus thymallus* populations. Our assessment is based on several population parameters including total and juvenile densities as well as their rate of change. This allows an in‐depth analysis of factors governing population dynamics, including density dependence and factors only operative at individual development stages or ages. Additionally, we incorporated possibly confounding factors such as predation, temperature, and hydrology into our analyses. These analyses are accompanied by a characterization of the flushing events with respect to sediment balance, SSC, and oxygen concentration, as well as a description of the sediment up‐ and downstream of reservoirs regarding its suitability for spawning.

## 2 MATERIALS AND METHODS

### 2.1 Study site

The Mur River is one of the largest rivers in Austria, with a total length of 444 km. It rises in the Hohe Tauern National Park (1,898 m above sea level) and about 325 km are within Austria. The Austrian catchment comprises approximately 10,341 km^2^.

The Mur River has a nivo‐pluvial hydrological regime with low discharge in winter, elevated flows during the snow melting period in spring and precipitation‐caused flood events in summer and early autumn. High water levels predominantly occur in summer (July, August). Extreme high water events (ca. HQ5) were observed in 1989, 2002, 2005, and 2012 (Supporting Information Data S1).

Although affected by several hydropower plants (HPPs) and partly embanked, the upper reach is still one of the least impacted large rivers in Austria (Brilly, Sraj, Vidmar, Horvat, & Koprivsek, 2012; Matulla, Schmutz, Melcher, Gerersdorfer, & Haas, 2007) and is considered one of the highest rated grayling sites in Austria from a fisherman's perspective. A compilation of salmonid density and biomass in 25 river stretches in Austria identified the Upper Mur River as the one with the second highest values concerning grayling biomass and density (Kaufmann et al., 1991). Recreational fisheries management includes stocking with brown trout (*Salmo trutta fario*) and rainbow trout (*Oncorhynchus mykiss*). Exploitation rates are usually low as most stretches are privately owned and fishing is restricted by license.

**Figure 1:**
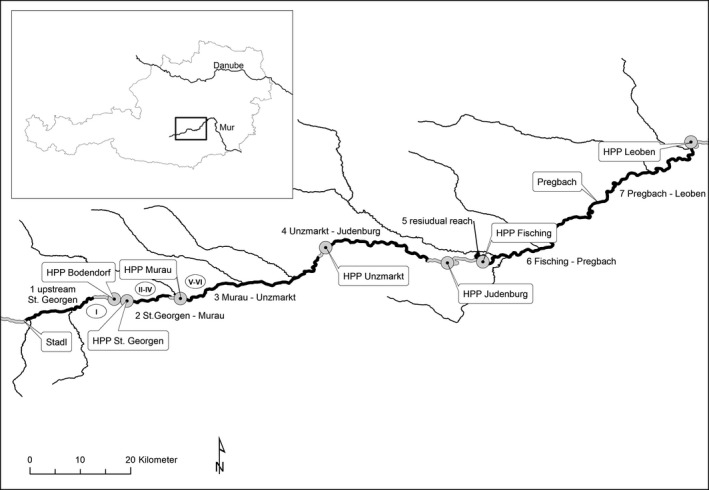
Study site between Stadl (r‐km 403) and Leoben (r‐km 270) with power plants (HPP), the different fishing stretches (black), and location of sediment samples (I–VI). The insert shows the location of the study site in Austria

The study site comprises a section of 133 km and is located between r‐km 403 (Stadl) and r‐km 270 (Leoben). HPPs are located upstream of Murau (HPPs Bodendorf and St. Georgen), at Murau, Unzmarkt, Judenburg, and Fisching (Figure 1). With the exception of HPP Fisching, all HPPs are standard run‐of‐river power plants. HPP Fisching is a run‐of‐river diversion hydropower plant, that is, water from the main river is diverted at r‐km 317. The residual flow section extends to the location of the powerhouse at r‐km 320.7. All HPPs have appropriate flood control devices, which can be used for flushing. HPP Murau and HPP Judenburg have overflow weirs and the sluice gates are used to drawdown the water level. All the other HPPs have radial gates (Table 1). During drawdown, the top of the sills of the radial gates are close to the river bed, so gravel can pass the weir efficiently.

**Table 1:** Characteristics of the different HPPs in the stretch between Stadl and Leoben

HPP	Type	Beginning of operation	Capacity (MW)	Reservoir length (km)	Weir gates
Bodendorf	Run‐of‐river plant	1982	7.0	2.4	Two radial gates
St. Georgen	Run‐of‐river plant	1985	6.0	1.6	Two radial gates
Murau	Run‐of‐river plant	1908	4.5	0.8	Overflow and sluice
Unzmarkt	Run‐of‐river plant	1989	4.8	2.4	Two radial gates
Judenburg	Chain of three run‐of‐river plants	1901–1911	6.2	3.1	Overflow and sluice
Fisching	Diversion plant	1994	21.9	4.5	Three radial gates

The section can be divided into stretches with differing hydromorphic characteristics. The most natural stretch is the one between Unzmarkt and Judenburg (mean hydromorphological status = 1.7, Table 2), and the most altered one lies between St. Georgen and Murau (mean hydromorphological status = 3.0, Table 2). Hydromorphological data were extracted from the “Digitaler Atlas Steiermark‐Gewässer & Wasserinformation” (2017), which is based on the Austrian manual for the assessment of the hydromorphological status (Mühlmann & Mauthner‐Weber, 2010).

**Table 2:** Hydromorphic characteristics of the different stretches between Stadl and Leoben; the last three columns give the mean values of the hydromorphological status of the stretches in a 5‐point scale (1‐natural, 2‐near natural, 3‐altered, 4‐shored, and 5‐artificial; Digitaler Atlas Steiermark‐Gewässer & Wasserinformation, 2017)

Stretch	r‐km	Meter above sea level	Mean river width (m)	*Q*_mean_ (m^3^/s)	River bed morphology	Bank morphology	Total morphology
1. Upstream St. Georgen	403.0–390.5	900–840	32	33	2.8	1.8	2.8
2. St. Georgen–Murau	386.0–378.0	840–800	32	34	3.0	2.0	3.0
3. Murau–Unzmarkt	377.0–351.5	800–720	41	36	2.6	1.2	2.6
4. Unzmarkt–Judenburg	349.0–330.0	720–700	44	46	1.7	1.2	1.7
5. Residual flow reach Fisching	321.0–317.5	680–660	56	9	2.1	2.0	2.1
6. Fisching–Pregbach	317.5–289.5	660–600	50	57	2.4	1.1	2.4
7. Pregbach–Leoben	289.5–270.0	600–540	50	79	2.5	1.1	2.5

The uppermost stretch is not affected by drawdown flushing and was thus used as a control in our analyses.

### 2.2 Sediment management at the Mur reservoirs

For the reservoirs in Bodendorf and Fisching, ecologically sustainable sediment management regimes have been developed beginning in the late 1990s by the operators in cooperation with planners, authority, and fishermen. Flushing events are initiated simultaneously at the power plants Bodendorf, St. Georgen, Murau, and Unzmarkt on the one hand, and Fisching and Judenburg on the other hand. Flushing in each of the two stretches is initiated when a remobilization of deposits (gravel, sand, and fines) can be expected based on a 2‐D hydraulic model of the reservoirs and the forecasted discharge. The schemes have been improved further during the ALPRESERV EU INTERREG IIIB project and now include recommendations for SSC and duration of flushing events, hydrological and seasonal restrictions, and postflushing with clear water in the residual flow reach. Details onto the regimes and their development are provided in Supporting Information Data S2.

### 2.3 Sampling design

In this study, the results of several investigations addressing fish ecology and reservoir management have been integrated. Most of the data have been gathered during monitoring programs to support authorities and the operators of the power plants in reservoir management and its assessment. A list of the data sources used in the study is provided in Supporting Information Data S3.

Our main objective was to assess the ecologically adjusted sediment management scheme. Such an assessment is not straightforward, as confounding effects (e.g., high water levels associated with high sediment load) may blur the results, especially in short‐term investigations. To disentangle these effects and to analyze population dynamics, long‐term investigations are necessary. To gain further insight into the processes structuring the grayling populations, we supplemented the fish data with information on river morphology, hydrology, temperature, and predator densities.

### 2.4 Discharge and temperature

Discharge and water temperature are routinely collected at the gauge Fisching. The gauge Fisching is situated in the middle of the study site, and values are representative for the whole stretch. We used mean daily values for statistical analyses. A description of the hydrograph and the temperature regime is given in Supporting Information Data S1.

### 2.5 Abiotic parameters during flushing events

SSC was measured downstream of the weirs with the Imhoff cone method, the gravimetric method, and turbidity meters. The Imhoff cone method is an indirect technique where the volume of settled sediment is read from the cones after allowing settling for 10 min. For a part of the samples, SSC was measured with gravimetric methods allowing the transformation of settleable solids to SSC by means of linear regression (Supporting Information Data S4). Similarly, a calibration check was performed during the flushing events for each turbidity meter to allow calculation of SSC.

Oxygen concentrations were measured during three flushing events in HPP Bodendorf and once in HPP Fisching with an oxygen meter.

### 2.6 Sediment retention and its removal during flushing operations

In the reservoirs Bodendorf and Fisching, topographical data are routinely collected using state of the art 3° single‐beam echosounder with GPS reference. Additionally, grain size distributions were analyzed once for three different strata in each impoundment (Table 3).

**Table 3:** Grain size distribution in the Bodendorf and Fisching reservoirs

	*d*_50_ (mm)			% Gravel (>2 mm)			Type of extraction	Year
	Upper part of reservoir	Middle part of reservoir	Lower part of reservoir	Upper part of reservoir	Middle part of reservoir	Lower part of reservoir		
Bodendorf	20–32	3–7	<1	85–90	20–50	5–10	Mechanical extraction	2004
Fisching	8–16	0.063–0.25	<0.01	85–90	0–10	0	Freeze core	2007

**Figure 2:**
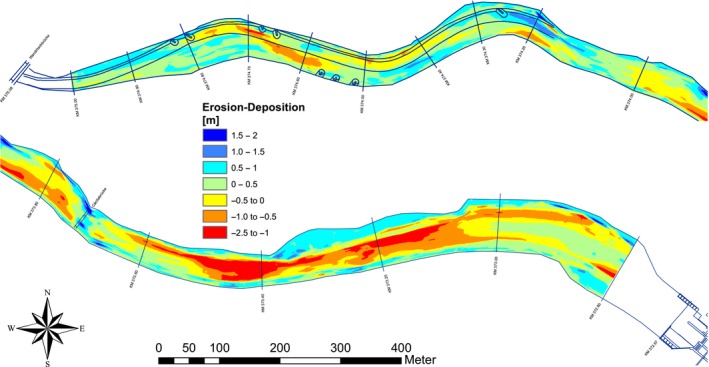
Bed level changes (difference map Oct 2005–May 2006) for the upper part of the reservoir Bodendorf, red indicates erosion, blue deposition

The echo sounding cross sections were used to generate Digital Terrain Models (DTMs). From the compiled DTMs from all available survey years, difference maps (Figure 2) were generated. These maps enable comparative volumetric analysis to determine change over a given period. Together with the information on the grain size distribution, the retention and removal of total sediment as well as the retention and removal of gravel (>2 mm grain size) were calculated.

### 2.7 Sediment data

In March 2005 and March 2006, a total of 44 freeze cores were taken at six sites down to a sediment depth of 100 cm. One site (nine samples) was located in the control section, three sites (nine, nine, and four samples) were located between St. Georgen and Murau, and two sites (nine and four samples) were situated between Murau and Unzmarkt (Figure 1). Six sediment layers (0–10, 10–20, 20–40, 40–60, 60–80, and 80–100 cm) were separated and analyzed. Sediment particle size distribution was determined for each depth layer by passing the material through a set of seven sieves (200, 63, 20, 6.3, 2, 0.63, and 0.2 mm). Each fraction was expressed as a percentage of total weight of sediment. The uppermost site is not affected by reservoirs and thus was used as a control. To characterize the suitability of the sediment for spawning, the median, the Q75 quartile, and the fine sediment content (<0.63 mm) were calculated.

**Figure 3:**
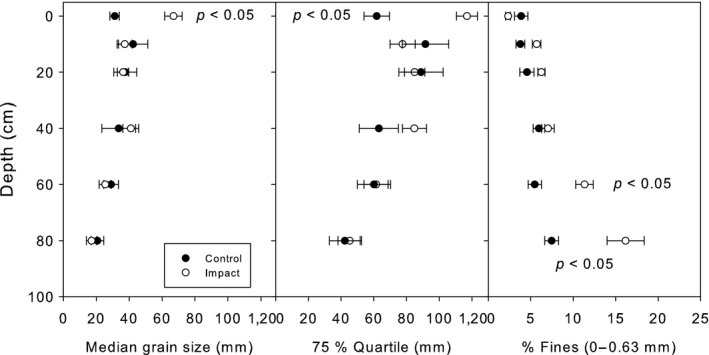
Sediment characteristics in the control reach and in the impacted reaches

The sediment was dominated by gravel (56.4%) and cobble (25.8%), sand and silt contributed to 17.7%. Overall sediment composition differed between depth strata (*p* < 0.05) and between control and impacted sites (*p* < 0.05). The uppermost layer was coarser in the sites downstream of the impoundments, indicating the existence of an armor layer (Figure 3). Additionally, the content of fines (<0.63 mm) was higher in the lower sediment layers.

### 2.8 Cormorant data

Data on the total cormorant abundances in Styria were provided by BirdLife (International Waterbird Census, IWC). Counts were undertaken yearly in January. Details onto the methodology can be found in Wetlands International (2010). In Styria, cormorant densities were low before the 1990s and increased thereafter. In 2001, maximum numbers were observed with more than 1,000 individuals. Since then, densities declined and varied between 100 and 500 individuals per census (Supporting Information Data S5).

### 2.9 Fish data

From 1990 to 2015, 140 autumnal electrofishing surveys were conducted in free‐flowing stretches along the Mur River. Fish densities were assessed using either two‐pass electrofishing or strip fishing (Schmutz, Zauner, Eberstaller, & Jungwirth, 2001). Fish captured were identified, measured (total length, TL), and then returned to the river. Abundance was assessed on a catch‐per‐unit‐effort basis (CPUE, fish per ha). For analysis, surveys were grouped based on the stretch and the survey date, respectively, resulting in 71 samples which were used for statistical analysis of total densities.

When length measurements were available, young of the year (YOY) were identified based on their size. These data were supplemented by electrofishing data from a sampling campaign in 2005 to 2006 (113 samples) which focused on YOY densities in autumn. A grouping of the data set based on the stretch and the survey date resulted in 67 samples which were used for statistical analysis of autumnal YOY densities.

Larval fish densities were assessed by Point Abundance Sampling (PAS) and expressed as catch‐per‐throw. Fishing took place from June/July to October/November each year between 2005 and 2012 in the stretch between Murau and Unzmarkt (stretch 3). In each year, 450 points were sampled at four to six dates in biweekly to monthly intervals. Fish were determined to species level and measured to the nearest mm TL. The data were used to analyze population dynamics of the YOY cohort.

Population change rate was calculated as

$$R=N_{t+1}/N_{t},$$

assuming a time step of 1 year, with *N_t_* = total number of grayling in year *t* and *N_t_*_+1_ = total number of grayling in the following year.

For larval fish, the intrinsic population change rate was calculated as

$$r=\left(\ln\left(N_{t1}\right)-\ln\left(N_{t0}\right)\right)/\left(t1-t0\right),$$

with *N_t_*_0_ = total number of grayling at *t*0 and *N_t_*_1_ = total number of grayling at *t*1; *t*0 and *t*1 correspond to the day number of the sampling date.

### 2.10 Statistical analyses

Statistical analyses were carried out in SPSS and in R 3.0.3. (R core Team, 2014) using the R packages glmulti (Calcagno, 2013) and MuMIN (Barton, 2016).

Generalized linear models (GLMs) were used to test for effects of biotic (cormorant densities, adult and total grayling abundance, fish size) and abiotic variables (discharge, temperature, drawdown) on total density, autumnal YOY abundance, larval densities, and rate of population change. We considered different hydrological and temperature variables (mean and maximum values) such as (a) data from May to October, which focus on the larval and juvenile phase of grayling, (b) data from April to October, which additionally consider the spawning and interstitial phase, (c) winter temperatures, and (d) the month/day when the maximum discharge occurred, reflecting underlying ecological processes supposed to affect grayling population dynamics. The river stretch was used as a categorial variable in the analyses. All possible combinations of predictor variables were calculated with the restriction that only one variable per model was considered of each variable group (Table 4) to avoid multicollinearity.

**Table 4:** Candidate models for the different dependent variables with predictor variables, their abbreviations and their descriptions

Dependent variable, data set	Predictors		
	Variable group/surrogate	Independent variable	Description
Total density (EF) and population change rate (EF)	Temperature	Tmean	Mean temperature between November and October
		Twinter	Mean temperature between November and February
		Tmin	Minimum temperature between November and October
	Discharge (Q)	Qmean	Mean discharge between November and October
		Qmax	Maximum discharge between November and October
	Bird predation	cormorants	Number of cormorants in January
	Grayling	denyear−1	Grayling density the year before sampling
	Flushing	flushing	Yes/no
	Stretch	stretch	Factor
YOY densities in autumn (EF)	Temperature	T4–10	Mean temperature between April and October
		T5–10	Mean temperature between May and October
	Discharge (Q)	Q4–10	Mean discharge between April and October
		Q5–10	Mean discharge between May and October
		Qmx4–10	Maximum discharge between April and October
		Qmx5–10	Maximum discharge between May and October
	Surrogate for fish Size	DQmx	The day at which the maximum discharge occurred
		MQmx	The month at which the maximum discharge occurred
	Grayling	adyear−1	Adult density the year before sampling
	Flushing	flushing	Yes/no
	Stretch	stretch	Factor
YOY densities in spring (PAS)	Temperature	T4–6	Mean temperature between April and June
	Discharge (Q)	Q4–6	Mean discharge between April and June
		Qmx4–6	Maximum discharge between April and June
	Flushing	flushing	Yes/no
YOY densities in autumn (PAS)	Temperature	T4–10	Mean temperature between April and October
		T5–10	Mean temperature between May and October
	Discharge (Q)	Q4–10	Mean discharge between April and October
		Q5–10	Mean discharge between May and October
		Qmx4–10	Maximum discharge between April and October
		Qmx5–10	Maximum discharge between May and October
	Fish size	DQmx	The day at which the maximum discharge occurred
		MQmx	The month at which the maximum discharge occurred
	Grayling	adyear−1	Adult density the year before sampling
	Flushing	flushing	Yes/no
Larval fish intrinsic population change rate (PAS)	Discharge (Q)	QmeanP	Mean discharge between two sampling occasions
		QmaxP	Maximum discharge between two sampling occasions
	FISH size	Smean	Mean larval size between sampling
		Send	Mean larval size at the end of the period
	Grayling	yoydens	YOY density on begin of the period
	Flushing	flushing	Yes/no

*Note.* EF: electrofishing; PAS: point abundance sampling; YOY: young of the year.

First analyses were performed with the data from the uppermost stretch that is not affected by HPPs (termed “control” or “upstream St. Georgen” throughout the text), to identify the variables supposedly regulating grayling dynamics under “natural” conditions. In a second set of analyses, all data were analyzed. Reduced data sets (i.e., data, where at least two consecutive years were available) were used to detect density dependence in population regulation. Individuals aged ≥3+ were designated as potential spawners (=adults).

Models were ranked according to their information content as determined by Akaike's information criterion corrected for small sample sizes (AIC_c_).

For all models that had an AIC_c _difference (∆AIC_c_) <2 from the best model (= model with lowest AIC_c_ value), we calculated their AIC_c_ weights. Higher AIC_c_ weights indicate a higher relative likelihood of a model compared with competing models (Symonds & Moussalli, 2011; Wagenmakers & Farrell, 2004).

### 2.11 Reservoir sedimentation & flushing operations

**Figure 4:**
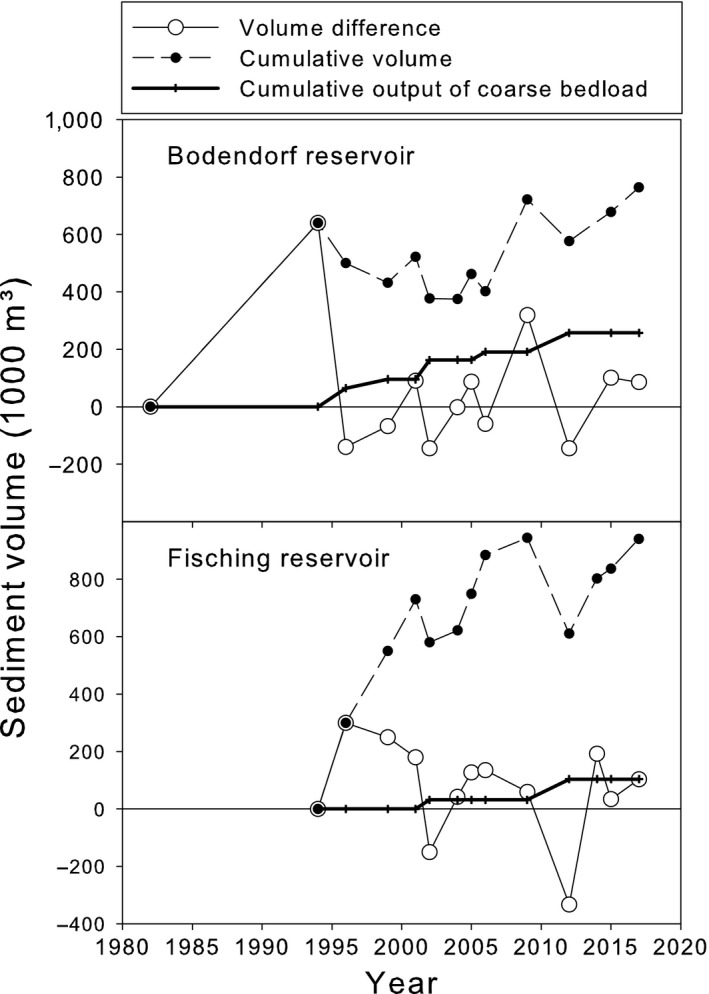
Trend in total sediment volume (zero = start of operation), volume difference between echo sounding surveys, and output of coarse bed load for Bodendorf and Fisching reservoirs

The yearly sedimentation rates were about 10% of the original storage volumes for both reservoirs (Figure 4). They corresponded to about 45,000 m^3^/year in Bodendorf with 20,700 m^3^ gravel (grain size > 2 mm) and 24,300 m^3^ fine sediments. For Fisching reservoir, sedimentation was estimated to approximate 80,000 m^3^/year with 17,200 m^3^ gravel and 62,800 m^3^ fines. The data for gravel relate to the mean yearly bed load upstream of the reservoirs assuming that all grain sizes > 2 mm are deposited.

Since the beginning of operation, seven flushing operations have been initiated at the power plant Bodendorf (together with power plants St. Georgen, Murau, and Unzmarkt) and six at the power plant Fisching. A strict coordination between the flushing operations at the two reservoirs was not possible due to slightly differing hydrological conditions in the catchment, the differing channel morphology, and different grain size distributions. Nevertheless, flushing at the two reservoirs happened concerted in most of the cases.

Additionally to fine sediments, considerable amounts of gravel and coarse sand (grain size > 2 mm) were remobilized. This bed load can be estimated to account for about 22% of total sediments in Fisching and 46% in Bodendorf.

The duration of the flushing events varied between 14.5 and 68.0 hr. Mean SSC varied between 0.6 and 9.7 g/L. Maximum values were mostly below 8 g/L, only once values exceeded 15 g/L for a limited time (Table 5).

**Table 5:** Flushing operations at the HPPs Fisching and Bodendorf and corresponding SSC and oxygen concentration; time specifications include reservoir level operations; Qd_max_—maximum discharge (daily mean) at Fisching during the flushing operation; samples were taken at Zeltweg (Fisching) and St. Georgen ob Murau (Bodendorf)

Site/year	Start time	End time	Qd_max_	*h*	SSC volumetric, turbidity meter			SSC gravimetric			Min O_2_	
					g/L mean	g/L max	N	g/L mean	g/L max	N	Mg/L	%
Bodendorf												
1996	13.05. 14:00	14.05. 23:00	155	33	9.7	17.9	n.a.					
1999	02.09. 11:00	04.09. 18:00	162	55	2.1	6.8	201				9.3	82
2002	11.08. 22:00	14.08. 18:00	382	68	2.3	7.8	146				9.1	86
2004	20.06. 08:00	21.06. 23:45	157	40	1.7	6.6	116				7.7	68
2006	20.05. 09:00	22.05. 00:30	235	40	1.9	4.1	60	2.0	7.2	60		
2012a^a^	15.07. 19:45	17.07. 14:30	331	43	0.6	3.6	37					
2012b^a^	21.07. 04:00	23.07. 00:00	350	44	1.0	5.2	39					
Fisching												
1999	23.07. 13:00	24.07. 10:00	241	21	1.6	5.9	89				9.9	91
2002	12.08. 14:00	14.08. 18:00	382	52	2.1	4.6	219	2.6	5.1	40		
2004^b^	20.06. 21:30	21.06. 11:30	157	14	1.5	3.8	81					
2009a,b	13.05. 08:00	14.05. 21:00	192	37	1.5	3.6	49	0.9	2.9	14		
2012a	15.07. 19:45	17.07. 14:30	331	43	2.7	7.2	135	2.6	6.7	135		
2012b^b^	21.07. 06:00	23.07. 19:15	350	61	1.9	2.5	114					

^a^ Measured with a turbidity meter. ^b^ Flushing was halted due to receding water levels.

At the three flushing events in Bodendorf where oxygen concentration was measured, no indication of oxygen depletion was evident (Table 5). In 1999, oxygen concentration was always above 9.3 mg/L (mean: 10.2 ± 0.4 *SD*). In 2002, mean oxygen concentration was 14.3 +1.8 *SD* mg/L with a minimum of 9.1 mg/L. In 2004, oxygen concentration varied between 7.7 and 13.8 mg/L (mean: 9.6 ± 1.0 *SD*) and oxygen saturation was always above 68%. At Fisching, data from 1999 also indicate sufficient oxygen supply during the drawdown (mean: 11.4 ± 0.8 *SD*, MIN = 9.9 mg/L).

## 3 RESULTS

### 3.1 Total grayling densities

**Figure 5:**
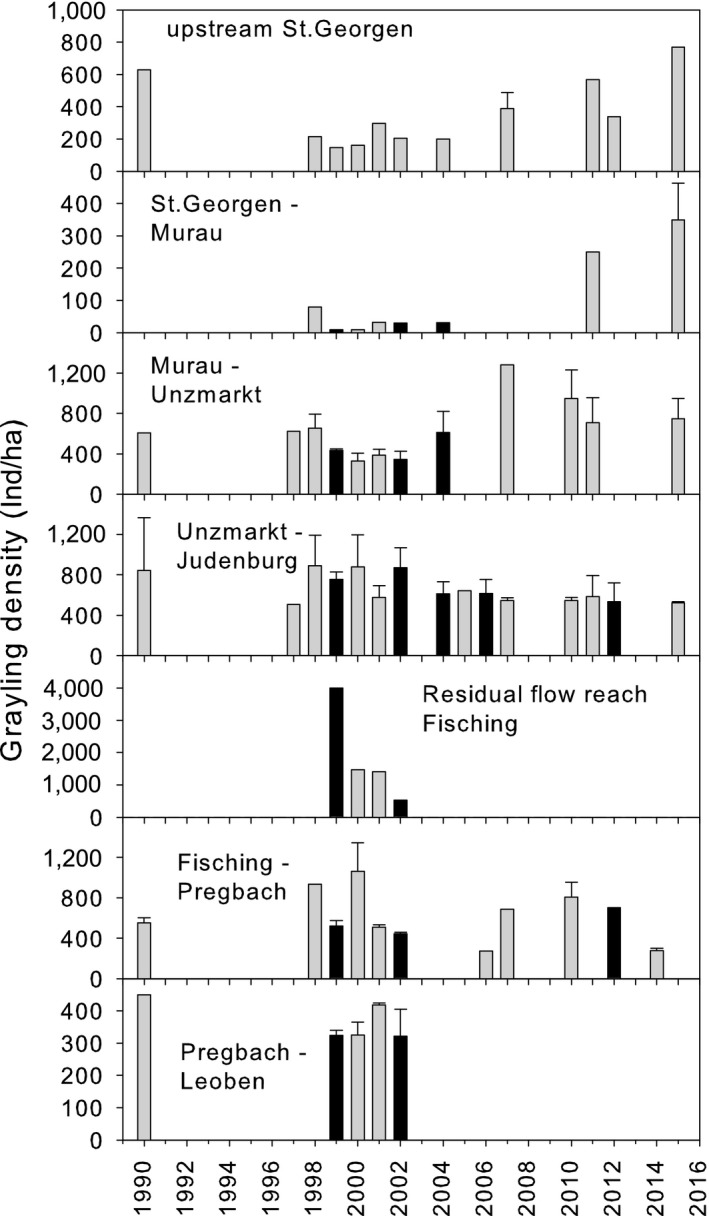
Autumnal densities (mean + *SE*) of grayling in different sections of the Mur River between 1990 and 2015. Black bars indicate flushing operations (including incomplete flushing due to receding water levels)

Densities of grayling varied considerably between the different sections and years. Maximal total densities (>3,000 grayling ha^−1^) were observed in the residual flow reach downstream of the HPP Fisching, generally low densities in the stretch between St. Georgen and Murau (Figure 5).

In the control reach, total densities were related to the yearly mean temperature and to the mean discharge. In the analysis of all data, only stretch and mean temperature had an effect on total densities. The analysis of the rate of population change revealed winter temperature, the densities in the year before sampling, and maximum discharge as the most influential variables. None of the best ranked models showed a negative effect of flushing (Table 6, Supporting Information Data S6).

**Table 6:** Results of model selection for total densities and population change rate

Nr.	AIC_c_	deltaAIC_c_	*ω* ***_i_***	Site	Dependent	Independent variables		
1	143.74	0.00	0.55	Control	Total density	Tmean		
2	144.18	0.43	0.45			Qmean	Tmean	
3	1,073.31	0.00	0.66	All	Total density	stretch		
4	1,074.64	1.34	0.34			stretch	Tmean	
5	16.60	0.00	1.00	Control	R	Only intercept		
6	62.43	0.00	0.53	All	R	Twinter	denyear−1	
7	63.74	1.31	0.27			Tmin	denyear−1	
8	64.37	1.95	0.20			Twinter	Qmax	denyear−1

### 3.2 YOY PAS surveys

The density of larvae at the first sampling date each year was negatively related to the maximum discharge in spring. The population change rate of fish larvae between two consecutive sampling events was negatively related to the discharge within this period and positively related to mean larval size. Autumnal densities were best explained by discharge and temperature. None of the best ranked models showed an effect of flushing on autumnal YOY abundance (Table 7, Supporting Information Data S6).

**Table 7:** Results of model selection for larval densities and larval fish intrinsic population change rate (stretch 3 Murau–Unzmarkt)

Nr.	AIC_c_	deltaAIC_c_	*ω* ***_i_***	Dependent	Independent variables	
9	37.87	0	0.71	YOY spring (PAS)	Only intercept	
10	39.69	1.82	0.29		Qmx4–6	
11	−131.11	0.00	0.52	YOY r (PAS)	QmeanP	Send
12	−130.94	0.17	0.48		QmeanP	Smean
13	17.22	0.00	0.38	YOY autumn (PAS)	Qmx4–10	T4–10
14	17.22	0.00	0.38		Qmx5–10	T4–10
15	18.16	0.93	0.24		T4–10	

### 3.3 YOY electrofishing surveys

**Figure 6:**
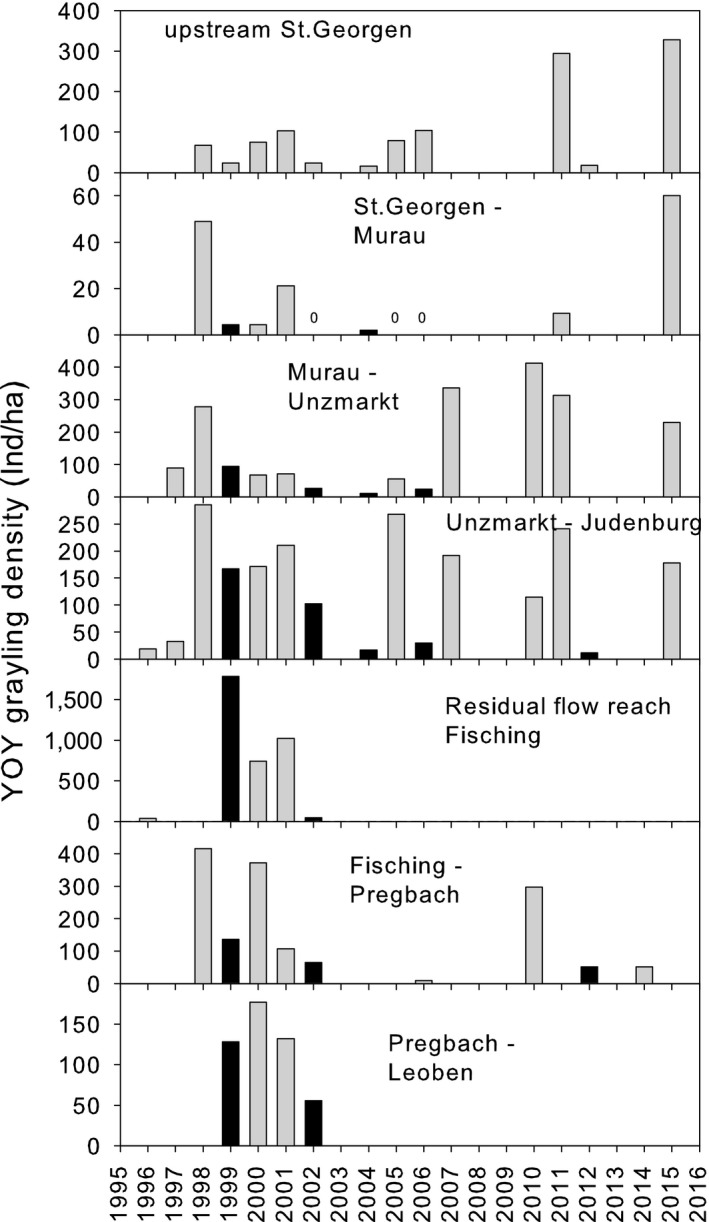
Autumnal YOY densities (mean) in different sections of the Mur River between 1996 and 2015. Zeros indicate samples with no fish. Black bars and underlined zeros indicate flushing operations

Autumnal abundance of YOY varied considerably between stretches. Highest densities were observed in the residual flow reach, and lowest densities were found in the stretch between St. Georgen and Murau. Generally, strong year classes coincided with dry years, weak ones with wet years which were often accompanied by flushing (Figure 6).

The analyses of the control site revealed hydrology and temperature as the most influential variables. The same was true for the complete data set. Of all tested variables, only site, temperature, maximum discharge, and the month/day when this discharge occurred were included in the best models. Temperature had a positive effect, and *Q*_max_ negatively affected autumnal YOY abundance. A flood had a less negative effect when it occurred late in the year. The analysis with a reduced data set, including potential spawners as a predictor, showed evidence for *Q*_max_ and stretch to influence autumnal YOY abundance. None of the analyses included flushing in the best models according to the AIC_c_ criterion (Table 8, Supporting Information Data S6).

**Table 8:** Results of model selection for autumnal YOY densities

Nr.	AIC_c_	deltaAIC_c_	*ω* ***_i_***	Site	Dependent	Independent variables			
16	130.21	0.00	0.28	Control	YOY autumn	Qmx4–10	T5–10		
17	130.25	0.04	0.28			Qmx5–10	T5–10		
18	130.67	0.46	0.22			Qmx4–10	T4–10		
19	130.71	0.50	0.22			Qmx5–10	T4–10		
20	921.06	0.00	0.10	All	YOY autumn	Stretch	Qmx5–10		
21	921.06	0.00	0.10			Stretch	Qmx4–10		
22	921.34	0.28	0.09			Stretch	Qmx5–10	DQmx	
23	921.39	0.33	0.09			Stretch	Qmx4–10	DQmx	
24	921.71	0.66	0.08			Stretch	Qmx5–10	MQmx	
25	921.76	0.70	0.07			stretch	Qmx4–10	MQmx	
26	922.08	1.03	0.06			Stretch	Qmx5–10	T4–10	DQmx
27	922.15	1.10	0.06			Stretch	Qmx4–10	T4–10	DQmx
28	922.19	1.13	0.06			Stretch			
29	922.43	1.37	0.05			Stretch	Qmx5–10	T4–10	
30	922.43	1.38	0.05			Stretch	Qmx4–10	T4–10	
31	922.60	1.54	0.05			Stretch	Qmx5–10	T4–10	MQmx
32	922.67	1.61	0.05			Stretch	Qmx4–10	T4–10	MQmx
33	922.93	1.87	0.04			Stretch	Qmx5–10	T5–10	DQmx
34	922.99	1.93	0.04			Stretch	Qmx4–10	T5–10	DQmx
35	411.30	0.00	0.50	All	YOY autumn (red)	Stretch	Qmx4–10		
36	411.30	0.00	0.50			Stretch	Qmx5–10		

## 4 DISCUSSION

### 4.1 Flushing events

Based on our analyses, no significant impact of flushing operations on population dynamics of European grayling was evident. If an effect is assumed, this effect was only minimal compared to other sources of variations.

Similarly, Espa et al. (2012) and Espa, Crosa, Gentili, Quadroni, and Petts (2015) found no adverse effect on the fish community of the Adda River (Italy), when flushing operations were ecologically adjusted and maximal sediment concentrations were 2.7 and 4.8 g/L, respectively, during the operation. Gutzmer, King, and Overhue (1996), Gutzmer, King, Overhue, and Chrisp (2002) reported minimal impacts to fish populations after operational adjustments of Spencer hydro sediment management. These results and comparable findings (e.g., Gerster & Rey, 1994) suggest that an ecologically adjusted reservoir management significantly reduces negative impacts on fish populations. Ecological adjustment often includes recommendations for SSC and duration of flushing events, as well as hydrological and seasonal restrictions. These adjustments were also undertaken at the Mur River reservoirs. Additionally, a postflushing with clear water was undertaken within the residual flow reach downstream of HPP Fisching to keep higher shear stresses and thus leading to a faster evacuation of sand and finer fractions. The measured values of SSC (overall mean: 2.3 ± 2.2 g/L; overall max: 6.1 ± 3.8 g/L) for a limited time (mean duration: 42 ± 14 hr; peak < 1 hr) seem to be within the tolerance limits of grayling. In the laboratory, salmonids generally tolerate SSC of >10 g/L for several days and SSC of >1 g/L over a month without significant mortality (e.g., Lake & Hinch, 1999, Michel, Schmidt‐Posthaus, & Burkhardt‐Holm, 2013). We also found no elevated fine sediment content in the uppermost sediment layers below reservoirs, and thus, long‐term effects on spawning habitats due to deposition of the flushed fine material seem to be insignificant.

In contrast to ecological adjusted flushing operations, the ones ignoring ecological consequences generally report much higher SSC values and/or were accompanied by low oxygen concentration. Repeatedly, this led to negative effects on fish and other biota (Supporting Information Data S7). Many studies report inconclusive results or only marginal effects. Brignoli, Espa, Quadroni, Torretta, and Ionescu (2015) for example assume a possible negative effect on fish densities in the discussion of their results, but state that these effects are of only marginal importance compared to seasonal variations.

A drawback of many studies is a flawed study design, that is, missing control sites; thus, results are often only indicative. For a sound statistical test of the effects of flushing operations, long‐term investigations or at least a BACI (Before‐After‐Control‐Impact) design are necessary. Without a control, natural fluctuating population parameters due to fishing mortality, predation, stocking, or high water events may be interpreted as effects of flushing operations. Flushing operations are often done during high flows. An attempt to disentangle the effects of displacement during floods and associated mortality from the effects of flushing was often not undertaken. Natural high flows may induce mortality or downstream displacement in stream fish populations (Carline & McCullough, 2003), especially in young salmonids (Ottaway & Clarke, 1981, Heggenes & Traaen, 1988, for a review see Warren, Dunbar, & Smith, 2015). During the flushing of Madesimo reservoir, parallel investigation in an affected reach and a second reach only affected by diluting flows showed that flow increases alone can disturb fish, suspended sediment increase had no additional effect on brown trout and bullhead (Quadroni et al., 2016).

Nevertheless, flushing operations without consideration of ecological consequences indisputable have negative impacts on aquatic habitats and species (Supporting Information Data S7).

### 4.2 Site specific differences

Our analyses revealed significant inter‐site variability, although this variability was smaller than the high temporal variability caused by floods. To depict spatial differences thoroughly, a more balanced database would have been necessary. Nevertheless, the data allow individual paired samples comparisons. These analyses indicate higher total densities in the stretches “04 Unzmarkt‐Judenburg,” “05 RW Fisching” and “06 Fisching‐Pregbach” and comparably lower ones in the control site and in stretch 02 “St.Georgen‐Murau.” YOY densities tend to be higher in “05 RW Fisching” and “07 Pregbach‐Leoben,” and lower in the control site and in stretch 02 “St.Georgen‐Murau” (Supporting Information Data S6). Overall, total and juvenile densities were related to the hydromorphological status of the reach (Spearman Rank Correlation; total: *r* = −0.86, *p* = 0.014; YOY: *r* = −0.71, *p* = 0.071. The high total and YOY densities in the residual flow reach “RW Fisching” may be explained by its high hydromorphological status together with a sufficient and “stable” flow; that is, the majority of the natural flow dynamics which may act as disturbances are more or less excluded from the reach. Nevertheless, high flows which induce morphological dynamics are still active allowing a nature‐like morphological development.

### 4.3 Cormorant predation

Our data showed no signs of predatory effects of Great Cormorant on grayling. This is in contrast to many studies blaming cormorants for reducing grayling populations (e.g., Steffens, 2011). Cormorants were also often cited as the most critical threat to grayling populations in Austria as well as in the Mur River (Woschitz & Parthl, 1997). The reasons for the low impact may be that—although there are night roosts nearby—only few individuals forage in the Upper Mur River, most cormorants feed in the reservoirs of the Lower Mur (e.g., Ringert, 2005). Furthermore, the average size of fish captured and ingested by Great Cormorant is significantly lower than the size at maturity. Cech and Vejrik (2011) reported a mean TL of preyed fish of 13.0 cm and fish ≤20 cm TL comprised more than 90% of the cormorants’ diet. This size class comprises to a large extent YOY, which are generally vulnerable to winter stressors (Hurst, 2007) and bird predation seems thus to work within the range of compensatory mortality.

### 4.4 Variable recruitment and density dependence

Our analyses did not support hypotheses related to flushing, but were consistent with patterns resulting from variable recruitment and density‐dependent effects. Two independent data sets (PAS, YOY) revealed hydrology and temperature as the main physical parameters affecting grayling recruitment. The same result was found by Charles, Mallet, and Persat (2006) in the Ain River. Their population model also revealed hydrology and temperature as the main variables affecting recruitment in European Grayling. Hydrology generally seems to be the main factor governing survival in early life history of salmonids in natural and nature‐like alpine streams. Unfer, Hauer, and Lautsch (2011) for example showed that high flows during incubation and emergence were negatively correlated with recruitment success of brown trout in the Ybbs River, a finding also supported by Cattanéo, Lamouroux, Breil, and Capra (2002) in their study on population dynamics of brown trout in 30 French stream reaches.

After the critical emergence and larval period, population dynamics are regulated by density‐dependent effects, that is, flow‐related losses early in ontogeny are compensated by increased growth or survival later in juvenile life history. These findings are consistent with Suter (1995) who found strong density dependence in grayling populations of the River Rhine. Density‐dependent compensatory processes have also been found in other potamodromous salmonids such as *Salmo marmoratus* (Vincenzi, Crivelli, Jesensek, Rubin, & Leo, 2007) and *Salmo trutta fario* (e.g., Richard, Cattanéo, & Rubin, 2015).

### 4.5 Spawning habitat

A highly important factor, in view of an ecologically adjusted flushing management, is the supply of gravel to the free‐flowing river stretches downstream from HPPs, as this enables the formation of new and loose uncolmated gravel substrates, which constitute a major prerequisite for the preservation of a river‐type‐specific fish and benthic fauna. This management strategy has already been proposed by Habersack (1996) for the Mur River who argued to flush material out of reservoirs during floods in an attempt to release material to the downstream section. Our data show that a significant amount of coarse sediments is released to downstream reaches during drawdown flushing. This sediment may improve habitat conditions especially with respect to spawning activities. Particle size distributions above and below reservoirs indicate suitable conditions for spawning with respect to the fine sediment content (mostly below 5% in the uppermost sediment layer). Nevertheless, the armored layer below reservoirs may hinder spawning to some extent. A regular supply of loose gravel should thus have positive effects on grayling populations.

### 4.6 General recommendations

Ecologically adjusted sediment management should be a prerequisite for reservoir operation. On the one hand, it may significantly reduce SSC and thus harmful impacts to fisheries during the operation, and on the other hand, it may deliver coarse substrate to downstream regions and thereby improving ecological conditions. Universally applicable technical recommendations to minimize negative impacts and maximize benefits cannot yet be given, as this depends on the species present, the hydrograph of the receiving stream and the type of reservoir. But some general recommendations should be considered: Generally, floods are a good opportunity to organize flushing, on the one hand permitting dilution of fine materials; on the other hand, SSC is generally high during high water events. Floods may and should be used to flush short‐term (daily to weekly) storage reservoirs. For long‐term (annual) storage basins, there is often no option and planning should focus on sensitive life stages of presumably impacted species (e.g., incubation period in salmonids, larval stages) and the hydrograph of the receiving stream.

When possible out‐flowing sediment loads should be regulated by hydraulic operations, two factors should be considered, the duration (including alternation of sediment releases and clear water) and the magnitude (peak and average allowable SSC) of sediment pulses.

### 4.7 Proposed management scheme for the Mur River

Generally, our study encourages the current reservoir management scheme.

Nevertheless, the problems related to the retention of gravel in years without flushing, with subsequent effects on spawning habitats, remain unsolved. The problem is related to seasonal and hydrological restrictions which prevent the use of every flood for flushing. For example, no flushing was initiated during the flood in October 2005 due to seasonal restriction (flushing is only permitted from April to September). In 2006, the hydrological restrictions allowed a flushing at HPP Bodendorf only, but not at HPP Fisching. These limitations led on the one hand to a discontinuous sediment management throughout the chain of reservoirs, on the other hand to extended periods without flushing especially at the HPP Fisching.

To overcome this shortcoming, we propose an adapted management scheme which aims at more frequent flushing. This may be accomplished by relaxing some seasonal and hydrological restrictions to achieve a periodic flushing regime in concordance with the hydrograph thereby mimicking the timing, magnitude, frequency, and duration of natural SSC pulses and gravel transport.

During small floods, we recommend drawdown routing (or sluicing) to minimize sand and silt deposition. Sluicing permits fine sediment to be transported through the reservoir rapidly to avoid sedimentation, and channel erosion may occur locally at the head of the reservoir. The required discharge and drawdown magnitude for sluicing depends on reservoir geometry and hydraulic parameters, in particular bed shear stress and particle size distribution.

During high flows—that is, whenever erosion can be expected—drawdown flushing involving extensive scouring and resuspending of sediments should be undertaken. In contrast to sluicing, whose aim is to pass sediment without allowing it to deposit, drawdown flushing focuses on scouring and resuspending deposited sediment and transporting it downstream. It involves the complete emptying of the reservoir to freely pass the flushing discharge through the dam without upstream impounding (Kondolf et al., 2014). Additionally, out‐flowing sediment loads should be regulated by reducing drawdown celerity during the transition phase of drawdown and free‐flow.

We suggest to dispense flushing from April to June when a drawdown flushing was performed the preceding year. The reason therefore is—although an effect of flushing on YOY was not evident according to model ranking—that eggs and larvae are often noted to be especially sensitive.

This flushing regime minimizes adverse downstream environmental impacts and maximizes benefits: A large proportion of the fines will be transported through the reservoir even at small floods so that the natural pattern of sediment discharge is approached. During large floods, flushing additionally allows for remobilization and transport of coarse material improving habitat conditions downstream through delivery of gravel.

## AUTHOR'S CONTRIBUTIONS

WR conceived and designed the study with substantial contributions of CS & HB. HB & WR organized and conducted data acquisition. CS conducted analyses with contribution of WR. WR wrote the first draft of the manuscript. All authors interpreted results, contributed to writing of the manuscript, and gave final approval for publication.

## Data Availability

Upon acceptance, all data supporting this study will be provided as Supporting Information accompanying this paper or be archived in an appropriate public archive.
