# Supplementary material for: Drawdown flushing in a chain of reservoirs—Effects on grayling populations and implications for sediment management
Source: Ecol Evol. 2019 Jan 15;9(3):1437–51. doi: 10.1002/ece3.4865 (PMC6374722; doi:10.1002/ece3.4865)
Supplement: Supplementary file 1 [file ECE3-9-1437-s001.docx]

**Drawdown flushing in a chain of reservoirs – effects on grayling populations and implications for sediment management**

**Supporting Information**

Walter Reckendorfer, VERBUND Hydro Power GmbH, Europaplatz 2, A-1150 Vienna, Austria, E-mail: walter.reckendorfer@verbund.com

Hannes Badura, VERBUND Hydro Power GmbH, Europaplatz 2, A-1150 Vienna, Austria, E-mail: Hannes.Badura@verbund.com

Claudia Schütz, Universität Wien, Department of Botany and Biodiversity Research, Rennweg 14, A-1030 Vienna, Austria, E -mail: claudia.schuetz@univie.ac.at

Corresponding Author: Walter Reckendorfer, T +43 (0)50313 – 51624

**Supporting Information Data S1**

**Discharge, hydrograph, and temperature in the River Mur**

The mean discharge of the Mur ranges from 33 m³s^-1^ at the gauge Bodendorf to 50 m³.s^-1^ at the gauge Fisching (Table S1_1). The Mur River has a nivo-pluvial hydrological regime with low discharges in winter, a snow melting period in spring and precipitation-caused flood events in summer and early autumn (Fig. S1_1, Fig. S1_2). Floods occur in the second half of the year from July to November but predominately in summer (Fig. S1_2).

| Discharge | Bodendorf | Fisching | Bruck/Mur |
| --- | --- | --- | --- |
| Q_95_ | 9 | 15 | 39 |
| MQ | 33 | 50 | 108 |
| HQ_1_ | 130 | 240 | 430 |
| HQ_5_ | 300 | 370 | 700 |
| HQ_10_ | 360 | 440 | 760 |
| HQ_30_ | 450 | 530 | 975 |
| HQ_50_ | 500 | 580 | 1,090 |
| HQ_100_ | 560 | 652 | 1,220 |

Table S1_1: Hydrological characteristics of the River Mur at Bodendorf (r-km 386), Fisching (r-km 321) and Bruck/Mur (r-km 245).

Long term mean daily water temperatures range from 1.1 to 16.9 C with highest values in July and August. The maximum value was recorded in August 2003 with 21.7 C (Fig. S1_3).

Fig. S1_1: Daily discharge at gauge “Fisching” from 01.01.1995 to 31.12.2015; HQ1 – yearly high water, HQ5 – flood with a recurrence interval of five years.

Fig. S1_2: Mean daily discharge (+SD) at gauge “Fisching”. X = discharges > HQ1 ” (period 01.01.1988 – 31.12.2015).

Fig. S1_3: Mean daily water temperature (+SD) at gauge “Fisching” (period 01.05.1998 – 31.12.2015).

**Supporting Information Data S2**

**Details on the reservoir management practices in the power plants Bodendorf and Fisching**

The sediment management schemes for the reservoirs at Bodendorf and Fisching include recommendations for SSC and duration of flushing events as well as hydrological and seasonal restrictions. Over the years the restrictions have been tightened and adjusted
(Table S2_1, Table S2_2, and Table S2_3).

At Bodendorf presently four seasons are defined with different hydrological restrictions. Furthermore, the decision if a drawdown flushing is undertaken depends on the flushing history. For instance, in the first year after a flushing event, a flushing is only undertaken in August and September at a discharge >130 m³s^-1^ or if the discharge exceeds 300 m³s^-1^ (Table S2_2).

At Fisching, flushing is presently restricted to the period between mid-May and end of October. Additionally, the discharge has to exceed 160 m³s^-1^. Only during large floods (> HQ20) a drawdown flushing is undertaken during the rest of the year (Table S2_3). After each flushing a post-flushing with clear water is undertaken within the residual reach downstream of HPP Fisching. Clear water post-flushing helps to keep higher shear stresses within the residual reach and thus leads to a faster evacuation of sand and finer fractions.

| Date | SSC max | season | Q_min_ | O_2min_ | Min. duration |
| --- | --- | --- | --- | --- | --- |
| Since 1995 | 20 ml L^-1^ | 12.05-30.09 | 100 m³s^-1^ (HQ_1_) | >5 mg L^-1^ | 2 days |
| Since 1998 | 15 ml L^-1^ | 12.05-30.09 | 80 m³s^-1^ | >5 mg L^-1^ | 2 days |
| Since 2005 | 15 ml L^-1^ | 15.04-15.05 | 80 m³s^-1^ (spring) | >5 mg L^-1^ | 2 days |
|  |  | 15.05-30.09 | 160 m³s^-1^ (summer) |  |  |
| Since 2007 | 15 ml L^-1^ | See Table S2_2 | See Table S2_2 | n.a. | 2 days |

Table S2_1: Development of the sediment management regime at Bodendorf; seasonal and hydrological restrictions, Q is given as discharge at power plant Bodendorf.

| Flushing | Season | Year 1 after a flushing | Year 2 after a flushing | Year 3 after a flushing | Year 4 after a flushing |
| --- | --- | --- | --- | --- | --- |
|  | 01.04-31.05 | No flushing allowed | >130 m³s^-1^ (HQ_1_) | >130 m³s^-1^ (HQ_1_) | >160 m³s^-1^ |
|  | 01.06-31.07 | No flushing allowed | No flushing allowed | No flushing allowed | >160 m³s^-1^ |
|  | 01.08-30.09 | >130 m³s^-1^ (HQ_1_) | >130 m³s^-1^ (HQ_1_) | >160 m³s^-1^ | >160 m³s^-1^ |
|  | Entire year | >300 m³s^-1^ (HQ_5_), throughout the entire year | | | |

Table S2_2: Sediment management regime at Bodendorf since 2007 - seasonal and hydrological restrictions; Q is given as discharge at power plant Bodendorf.

| Date | SSC max | season | Q_min_ | O_2min_ |
| --- | --- | --- | --- | --- |
| Since 1992 |  | 15.05-30.09 | 230 m³s^-1^ (HQ_1_) | n.a. |
| Since 1998 |  | 15.05-30.09 | 160 m³s^-1^ | n.a. |
| Since 2000 | 25 ml L^-1^ | 15.05-30.09 | 160 m³s^-1^ | n.a. |
| Since 2009 | n.a. | n.a. | 120 m³s^-1^ | n.a. |
| Since 2011 | 25 ml L^-1^ | 15.05-31.10  Entire year | 160 m³s^-1^  >500 m³s^-1^(HQ_20_) | n.a. |

Table S2_3: Development of the sediment management regime at Fisching. Q is given as discharge at power plant Fisching.

**Supporting Information Data S3**

**Data sources and description**

In this study, the results of several investigations addressing fish ecology and reservoir management have been integrated. Most of the data have been gathered during monitoring programs to support authorities and the operators of the power plants in reservoir management and its assessment. A list of the data sources used in the study is provided in Table S3_1.

Total densities and autumnal YOY densities were assessed using either two-pass electrofishing or strip fishing. Abundance was assessed on a catch-per-unit-effort basis (CPUE, fish.ha^−1^). Larval fish densities were assessed by Point Abundance Sampling (PAS) and expressed as catch-per-throw.

For analysis, the different surveys were grouped based on the stretch and the survey date respectively resulting in 71 samples which were used for statistical analysis of total densities, 67 samples which were used for statistical analysis of autumnal YOY densities, and 42 samples which were used for statistical analysis of larval densities (Table_S3_2).

Table S3_1: Data sources.

Table S3_2: Number of samples in each stretch. * No information on size classes available.

**Supporting Information Data S4**

**Transformation of settleable solids to suspended sediment concentration (SSC)**

SSC and settled sediment (Imhoff cone) are significantly related and can be described by the linear relationship y = 0.783 + 0.214*x for Fisching and y = 0.412 + 0.230*x for Bodendorf (Fig. S4_1).

Fig. S4_1: Relationship between settled sediment (Imhoff cone) and SSC. Regression lines for the two reservoirs.

**Supporting Information Data S5**

**Cormorant densities in Styria**

In Styria cormorants were rare winter visitors and passage migrants until the end of the 1980ies. From the beginning of the 1990ies on, higher numbers were counted. Maximum densities of more than 1000 cormorants were found in the beginning of the new century. Since then densities stabilized between 100 and 500 individuals with large inter-annual variation (Fig. S5_1).

Fig. S5_1: Number of cormorants (January) counted during the IWC in Styria and running mean (d= 3 years)

**Supporting Information Data S6**

**Detailed statistics**

Table S6_1 shows the detailed statistics (number of samples, coefficient estimates, standard error of coefficients, T values and significance) of the final models with AIC_c_ difference (∆AIC_c_) <2 from the best model (= model with lowest AIC_c_ value).

| **data set** | **dependent** | **sites** | **N** | **Nr.** | **Parameter** | **Coefficient** | **SE** | **T** | **Sig.** |
| --- | --- | --- | --- | --- | --- | --- | --- | --- | --- |
| electrofishing | total density | control | 11 | 1 | Intercept | -2339,2806 | 578,1833 | -4,05 | 0,003 |
|  |  |  |  |  | Tmean | 322,8838 | 69,1172 | 4,67 | 0,001 |
|  |  |  |  | 2 | Intercept | -3275,3632 | 665,4554 | -4,92 | 0,001 |
|  |  |  |  |  | Tmean | 372,9376 | 63,5872 | 5,86 | 0,000 |
|  |  |  |  |  | Qmean | 11,0759 | 5,2899 | 2,09 | 0,070 |
| electrofishing | total density | all | 71 | 3 | Intercept | 367,3540 | 192,0396 | 1,913 | 0,060 |
|  |  |  |  |  | 01 Stadl-St.Georgen | -10,9069 | 231,6085 | -,047 | 0,963 |
|  |  |  |  |  | 02 St.Georgen - Murau | -268,4088 | 244,8034 | -1,096 | 0,277 |
|  |  |  |  |  | 03 Murau-Unzmarkt | 272,3061 | 228,5729 | 1,191 | 0,238 |
|  |  |  |  |  | 04 Unzmarkt-Judenburg | 288,5213 | 220,0090 | 1,311 | 0,194 |
|  |  |  |  |  | 05 RW Fisching | 1027,4793 | 260,0229 | 3,951 | 0,000 |
|  |  |  |  |  | 06 Fisching-Pregbach | 191,2297 | 225,9724 | ,846 | 0,401 |
|  |  |  |  |  | 07 Pregbach-Leoben | 0^a^ |  |  |  |
|  |  |  |  | 4 | Intercept | -509,3069 | 837,0512 | -,608 | 0,545 |
|  |  |  |  |  | 01 Stadl-St.Georgen | -44,0917 | 233,3708 | -,189 | 0,851 |
|  |  |  |  |  | 02 St.Georgen - Murau | -295,1192 | 245,7595 | -1,201 | 0,234 |
|  |  |  |  |  | 03 Murau-Unzmarkt | 244,1245 | 229,7895 | 1,062 | 0,292 |
|  |  |  |  |  | 04 Unzmarkt-Judenburg | 272,4225 | 220,2473 | 1,237 | 0,221 |
|  |  |  |  |  | 05 RW Fisching | 1050,1540 | 260,5568 | 4,030 | 0,000 |
|  |  |  |  |  | 06 Fisching-Pregbach | 178,3217 | 226,0132 | ,789 | 0,433 |
|  |  |  |  |  | 07 Pregbach-Leoben | 0^a^ |  |  |  |
|  |  |  |  |  | Tmean | 108,9778 | 101,2853 | 1,076 | 0,286 |
| electrofishing | R | control | 5 | 5 | Intercept |  |  |  |  |
| electrofishing | R | all | 38 | 6 | Intercept | 0,3893 | 0,3004 | 1,296 | 0,203 |
|  |  |  |  |  | denyear-1 | -0,0003 | 0,0001 | -2,292 | 0,028 |
|  |  |  |  |  | Twinter | 0,3676 | 0,1221 | 3,010 | 0,005 |
|  |  |  |  | 7 | Intercept | 0,8512 | 0,1792 | 4,751 | 0,000 |
|  |  |  |  |  | denyear-1 | -0,0003 | 0,0001 | -2,153 | 0,038 |
|  |  |  |  |  | Tmin | 0,4222 | 0,1535 | 2,750 | 0,009 |
|  |  |  |  | 8 | Intercept | 0,7038 | 0,4947 | 1,423 | 0,164 |
|  |  |  |  |  | denyear-1 | -0,0003 | 0,0001 | -2,371 | 0,024 |
|  |  |  |  |  | Twinter | 0,3243 | 0,1341 | 2,419 | 0,021 |
|  |  |  |  |  | Qmax | -0,0009 | 0,0011 | -,803 | 0,428 |
| PAS | YOY density spring | stretch 3 | 8 | 9 | Intercept |  |  |  |  |
|  |  |  |  | 10 | Intercept | 6,6099 | 2,1379 | 3,092 | 0,021 |
|  |  |  |  |  | Qmx4-6 | -0,0251 | 0,0132 | -1,902 | 0,106 |
| PAS | YOY r | stretch 3 | 34 | 11 | Intercept | -0,0492 | 0,0269 | -1,829 | 0,077 |
|  |  |  |  |  | QmeanP | -0,0006 | 0,0003 | -2,262 | 0,031 |
|  |  |  |  |  | Send | 0,0007 | 0,0002 | 2,957 | 0,006 |
|  |  |  |  | 12 | Intercept | -0,0452 | 0,0260 | -1,738 | 0,092 |
|  |  |  |  |  | QmeanP | -0,0006 | 0,0003 | -2,214 | 0,034 |
|  |  |  |  |  | Smean | 0,0007 | 0,0002 | 2,923 | 0,006 |
| PAS | YOY density autumn | stretch 3 | 8 | 13 | Intercept | -7,482 | 1,807 | -4,140 | 0,009 |
|  |  |  |  |  | Qmx4-10 | -,005 | ,001 | -3,597 | 0,016 |
|  |  |  |  |  | T4-10 | ,741 | ,140 | 5,302 | 0,003 |
|  |  |  |  | 14 | Intercept | -7,482 | 1,807 | -4,140 | 0,009 |
|  |  |  |  |  | T4-10 | ,741 | ,140 | 5,302 | 0,003 |
|  |  |  |  |  | Qmx5-10 | -,005 | ,001 | -3,597 | 0,016 |
|  |  |  |  | 15 | Intercept | -9,818 | 2,916 | -3,367 | 0,015 |
|  |  |  |  |  | T4-10 | ,837 | ,237 | 3,528 | 0,012 |
| electrofishing | YOY density autumn | control | 9 | 16 | Intercept | -911,021 | 316,846 | -2,875 | 0,021 |
|  |  |  |  |  | Qmx4-10 | -,623 | ,191 | -3,258 | 0,012 |
|  |  |  |  |  | T5-10 | 90,938 | 23,956 | 3,796 | 0,005 |
|  |  |  |  | 17 | Intercept | -913,847 | 317,245 | -2,881 | 0,020 |
|  |  |  |  |  | T5-10 | 91,109 | 23,995 | 3,797 | 0,005 |
|  |  |  |  |  | Qmx5-10 | -,620 | ,191 | -3,247 | 0,012 |
|  |  |  |  | 18 | Intercept | -870,883 | 316,684 | -2,750 | 0,025 |
|  |  |  |  |  | Qmx4-10 | -,615 | ,196 | -3,141 | 0,014 |
|  |  |  |  |  | T4-10 | 92,574 | 25,214 | 3,672 | 0,006 |
|  |  |  |  | 19 | Intercept | -873,660 | 317,050 | -2,756 | 0,025 |
|  |  |  |  |  | T4-10 | 92,750 | 25,252 | 3,673 | 0,006 |
|  |  |  |  |  | Qmx5-10 | -,613 | ,196 | -3,130 | 0,014 |
| electrofishing | YOY density autumn | all | 67 | 20 | Intercept | 266,841 | 131,477 | 2,030 | 0,047 |
|  |  |  |  |  | Qmx5-10 | -,595 | ,320 | -1,858 | 0,068 |
|  |  |  |  |  | 01 Stadl-St.Georgen | -29,403 | 124,320 | -,237 | 0,814 |
|  |  |  |  |  | 02 St.Georgen - Murau | -125,090 | 126,186 | -,991 | 0,326 |
|  |  |  |  |  | 03 Murau-Unzmarkt | 10,914 | 122,148 | ,089 | 0,929 |
|  |  |  |  |  | 04 Unzmarkt-Judenburg | -4,142 | 120,068 | -,034 | 0,973 |
|  |  |  |  |  | 05 RW Fisching | 593,306 | 142,823 | 4,154 | 0,000 |
|  |  |  |  |  | 06 Fisching-Pregbach | 38,643 | 127,881 | ,302 | 0,764 |
|  |  |  |  |  | 07 Pregbach-Leoben | 0^a^ |  |  |  |
|  |  |  |  | 21 | Intercept | 267,529 | 131,706 | 2,031 | 0,047 |
|  |  |  |  |  | Qmx4-10 | -,596 | ,321 | -1,858 | 0,068 |
|  |  |  |  |  | 01 Stadl-St.Georgen | -29,702 | 124,329 | -,239 | 0,812 |
|  |  |  |  |  | 02 St.Georgen - Murau | -125,386 | 126,199 | -,994 | 0,324 |
|  |  |  |  |  | 03 Murau-Unzmarkt | 10,570 | 122,167 | ,087 | 0,931 |
|  |  |  |  |  | 04 Unzmarkt-Judenburg | -4,497 | 120,084 | -,037 | 0,970 |
|  |  |  |  |  | 05 RW Fisching | 593,200 | 142,827 | 4,153 | 0,000 |
|  |  |  |  |  | 06 Fisching-Pregbach | 38,386 | 127,886 | ,300 | 0,765 |
|  |  |  |  |  | 07 Pregbach-Leoben | 0^a^ |  |  |  |
|  |  |  |  | 22 | Intercept | 196,132 | 138,621 | 1,415 | 0,162 |
|  |  |  |  |  | Qmx5-10 | -,999 | ,418 | -2,390 | 0,020 |
|  |  |  |  |  | DQmx | 1,004 | ,677 | 1,483 | 0,144 |
|  |  |  |  |  | 01 Stadl-St.Georgen | -49,838 | 123,845 | -,402 | 0,689 |
|  |  |  |  |  | 02 St.Georgen - Murau | -148,397 | 125,908 | -1,179 | 0,243 |
|  |  |  |  |  | 03 Murau-Unzmarkt | -8,206 | 121,612 | -,067 | 0,946 |
|  |  |  |  |  | 04 Unzmarkt-Judenburg | -20,168 | 119,357 | -,169 | 0,866 |
|  |  |  |  |  | 05 RW Fisching | 593,281 | 141,394 | 4,196 | 0,000 |
|  |  |  |  |  | 06 Fisching-Pregbach | 21,211 | 127,146 | ,167 | 0,868 |
|  |  |  |  |  | 07 Pregbach-Leoben | 0^a^ |  |  |  |
|  |  |  |  | 23 | Intercept | 197,734 | 138,824 | 1,424 | 0,160 |
|  |  |  |  |  | DQmx | ,991 | ,675 | 1,468 | 0,147 |
|  |  |  |  |  | Qmx4-10 | -,994 | ,417 | -2,380 | 0,021 |
|  |  |  |  |  | 01 Stadl-St.Georgen | -50,042 | 123,904 | -,404 | 0,688 |
|  |  |  |  |  | 02 St.Georgen - Murau | -148,534 | 125,971 | -1,179 | 0,243 |
|  |  |  |  |  | 03 Murau-Unzmarkt | -8,464 | 121,679 | -,070 | 0,945 |
|  |  |  |  |  | 04 Unzmarkt-Judenburg | -20,498 | 119,423 | -,172 | 0,864 |
|  |  |  |  |  | 05 RW Fisching | 593,137 | 141,448 | 4,193 | 0,000 |
|  |  |  |  |  | 06 Fisching-Pregbach | 21,027 | 127,202 | ,165 | 0,869 |
|  |  |  |  |  | 07 Pregbach-Leoben | 0^a^ |  |  |  |
|  |  |  |  | 24 | Intercept | 192,400 | 141,469 | 1,360 | 0,179 |
|  |  |  |  |  | Qmx5-10 | -,930 | ,402 | -2,315 | 0,024 |
|  |  |  |  |  | MQmx | 25,887 | 18,971 | 1,365 | 0,178 |
|  |  |  |  |  | 01 Stadl-St.Georgen | -48,781 | 124,236 | -,393 | 0,696 |
|  |  |  |  |  | 02 St.Georgen - Murau | -147,463 | 126,343 | -1,167 | 0,248 |
|  |  |  |  |  | 03 Murau-Unzmarkt | -8,658 | 122,111 | -,071 | 0,944 |
|  |  |  |  |  | 04 Unzmarkt-Judenburg | -20,597 | 119,809 | -,172 | 0,864 |
|  |  |  |  |  | 05 RW Fisching | 592,713 | 141,792 | 4,180 | 0,000 |
|  |  |  |  |  | 06 Fisching-Pregbach | 21,135 | 127,604 | ,166 | 0,869 |
|  |  |  |  |  | 07 Pregbach-Leoben | 0^a^ |  |  |  |
|  |  |  |  | 25 | Intercept | 194,061 | 141,682 | 1,370 | 0,176 |
|  |  |  |  |  | MQmx | 25,507 | 18,905 | 1,349 | 0,183 |
|  |  |  |  |  | Qmx4-10 | -,925 | ,401 | -2,306 | 0,025 |
|  |  |  |  |  | 01 Stadl-St.Georgen | -48,928 | 124,293 | -,394 | 0,695 |
|  |  |  |  |  | 02 St.Georgen - Murau | -147,535 | 126,402 | -1,167 | 0,248 |
|  |  |  |  |  | 03 Murau-Unzmarkt | -8,832 | 122,176 | -,072 | 0,943 |
|  |  |  |  |  | 04 Unzmarkt-Judenburg | -20,848 | 119,872 | -,174 | 0,863 |
|  |  |  |  |  | 05 RW Fisching | 592,593 | 141,845 | 4,178 | 0,000 |
|  |  |  |  |  | 06 Fisching-Pregbach | 21,012 | 127,658 | ,165 | 0,870 |
|  |  |  |  |  | 07 Pregbach-Leoben | 0^a^ |  |  |  |
|  |  |  |  | 26 | Intercept | -462,354 | 504,801 | -,916 | 0,364 |
|  |  |  |  |  | Qmx5-10 | -1,015 | ,415 | -2,445 | 0,018 |
|  |  |  |  |  | T4-10 | 54,637 | 40,298 | 1,356 | 0,181 |
|  |  |  |  |  | DQmx | 1,138 | ,679 | 1,674 | 0,100 |
|  |  |  |  |  | 01 Stadl-St.Georgen | -68,814 | 123,754 | -,556 | 0,580 |
|  |  |  |  |  | 02 St.Georgen - Murau | -163,493 | 125,503 | -1,303 | 0,198 |
|  |  |  |  |  | 03 Murau-Unzmarkt | -22,267 | 121,187 | -,184 | 0,855 |
|  |  |  |  |  | 04 Unzmarkt-Judenburg | -32,927 | 118,877 | -,277 | 0,783 |
|  |  |  |  |  | 05 RW Fisching | 603,119 | 140,570 | 4,291 | 0,000 |
|  |  |  |  |  | 06 Fisching-Pregbach | 3,463 | 126,914 | ,027 | 0,978 |
|  |  |  |  |  | 07 Pregbach-Leoben | 0^a^ |  |  |  |
|  |  |  |  | 27 | Intercept | -457,886 | 505,114 | -,907 | 0,368 |
|  |  |  |  |  | T4-10 | 54,395 | 40,317 | 1,349 | 0,183 |
|  |  |  |  |  | DQmx | 1,122 | ,677 | 1,658 | 0,103 |
|  |  |  |  |  | Qmx4-10 | -1,008 | ,415 | -2,432 | 0,018 |
|  |  |  |  |  | 01 Stadl-St.Georgen | -68,899 | 123,829 | -,556 | 0,580 |
|  |  |  |  |  | 02 St.Georgen - Murau | -163,514 | 125,581 | -1,302 | 0,198 |
|  |  |  |  |  | 03 Murau-Unzmarkt | -22,416 | 121,270 | -,185 | 0,854 |
|  |  |  |  |  | 04 Unzmarkt-Judenburg | -33,163 | 118,958 | -,279 | 0,781 |
|  |  |  |  |  | 05 RW Fisching | 602,942 | 140,646 | 4,287 | 0,000 |
|  |  |  |  |  | 06 Fisching-Pregbach | 3,384 | 126,987 | ,027 | 0,979 |
|  |  |  |  |  | 07 Pregbach-Leoben | 0^a^ |  |  |  |
|  |  |  |  | 28 | Intercept | 123,250 | 108,528 | 1,136 | 0,261 |
|  |  |  |  |  | 01 Stadl-St.Georgen | -20,068 | 126,733 | -,158 | 0,875 |
|  |  |  |  |  | 02 St.Georgen - Murau | -108,350 | 128,412 | -,844 | 0,402 |
|  |  |  |  |  | 03 Murau-Unzmarkt | 31,519 | 124,106 | ,254 | 0,800 |
|  |  |  |  |  | 04 Unzmarkt-Judenburg | 12,817 | 122,144 | ,105 | 0,917 |
|  |  |  |  |  | 05 RW Fisching | 603,550 | 145,605 | 4,145 | 0,000 |
|  |  |  |  |  | 06 Fisching-Pregbach | 44,194 | 130,434 | ,339 | 0,736 |
|  |  |  |  |  | 07 Pregbach-Leoben | 0^a^ |  |  |  |
|  |  |  |  | 29 | Intercept | -265,957 | 498,561 | -,533 | 0,596 |
|  |  |  |  |  | Qmx5-10 | -,564 | ,321 | -1,759 | 0,084 |
|  |  |  |  |  | T4-10 | 44,849 | 40,487 | 1,108 | 0,273 |
|  |  |  |  |  | 01 Stadl-St.Georgen | -42,747 | 124,665 | -,343 | 0,733 |
|  |  |  |  |  | 02 St.Georgen - Murau | -134,935 | 126,257 | -1,069 | 0,290 |
|  |  |  |  |  | 03 Murau-Unzmarkt | 1,460 | 122,212 | ,012 | 0,991 |
|  |  |  |  |  | 04 Unzmarkt-Judenburg | -12,864 | 120,096 | -,107 | 0,915 |
|  |  |  |  |  | 05 RW Fisching | 601,385 | 142,735 | 4,213 | 0,000 |
|  |  |  |  |  | 06 Fisching-Pregbach | 25,979 | 128,147 | ,203 | 0,840 |
|  |  |  |  |  | 07 Pregbach-Leoben | 0^a^ |  |  |  |
|  |  |  |  | 30 | Intercept | -264,512 | 498,808 | -,530 | 0,598 |
|  |  |  |  |  | T4-10 | 44,774 | 40,493 | 1,106 | 0,273 |
|  |  |  |  |  | Qmx4-10 | -,565 | ,322 | -1,757 | 0,084 |
|  |  |  |  |  | 01 Stadl-St.Georgen | -43,002 | 124,676 | -,345 | 0,731 |
|  |  |  |  |  | 02 St.Georgen - Murau | -135,188 | 126,273 | -1,071 | 0,289 |
|  |  |  |  |  | 03 Murau-Unzmarkt | 1,165 | 122,234 | ,010 | 0,992 |
|  |  |  |  |  | 04 Unzmarkt-Judenburg | -13,174 | 120,115 | -,110 | 0,913 |
|  |  |  |  |  | 05 RW Fisching | 601,277 | 142,745 | 4,212 | 0,000 |
|  |  |  |  |  | 06 Fisching-Pregbach | 25,760 | 128,155 | ,201 | 0,841 |
|  |  |  |  |  | 07 Pregbach-Leoben | 0^a^ |  |  |  |
|  |  |  |  | 31 | Intercept | -445,191 | 506,578 | -,879 | 0,383 |
|  |  |  |  |  | T4-10 | 52,886 | 40,368 | 1,310 | 0,195 |
|  |  |  |  |  | Qmx5-10 | -,936 | ,399 | -2,343 | 0,023 |
|  |  |  |  |  | MQmx | 29,125 | 19,017 | 1,532 | 0,131 |
|  |  |  |  |  | 01 Stadl-St.Georgen | -66,941 | 124,252 | -,539 | 0,592 |
|  |  |  |  |  | 02 St.Georgen - Murau | -161,871 | 126,051 | -1,284 | 0,204 |
|  |  |  |  |  | 03 Murau-Unzmarkt | -22,254 | 121,807 | -,183 | 0,856 |
|  |  |  |  |  | 04 Unzmarkt-Judenburg | -32,941 | 119,448 | -,276 | 0,784 |
|  |  |  |  |  | 05 RW Fisching | 602,166 | 141,109 | 4,267 | 0,000 |
|  |  |  |  |  | 06 Fisching-Pregbach | 4,010 | 127,495 | ,031 | 0,975 |
|  |  |  |  |  | 07 Pregbach-Leoben | 0^a^ |  |  |  |
|  |  |  |  | 32 | Intercept | -440,973 | 506,915 | -,870 | 0,388 |
|  |  |  |  |  | T4-10 | 52,668 | 40,387 | 1,304 | 0,197 |
|  |  |  |  |  | MQmx | 28,697 | 18,951 | 1,514 | 0,135 |
|  |  |  |  |  | Qmx4-10 | -,929 | ,399 | -2,330 | 0,023 |
|  |  |  |  |  | 01 Stadl-St.Georgen | -66,978 | 124,322 | -,539 | 0,592 |
|  |  |  |  |  | 02 St.Georgen - Murau | -161,835 | 126,123 | -1,283 | 0,205 |
|  |  |  |  |  | 03 Murau-Unzmarkt | -22,322 | 121,884 | -,183 | 0,855 |
|  |  |  |  |  | 04 Unzmarkt-Judenburg | -33,100 | 119,525 | -,277 | 0,783 |
|  |  |  |  |  | 05 RW Fisching | 602,018 | 141,181 | 4,264 | 0,000 |
|  |  |  |  |  | 06 Fisching-Pregbach | 3,987 | 127,563 | ,031 | 0,975 |
|  |  |  |  |  | 07 Pregbach-Leoben | 0^a^ |  |  |  |
|  |  |  |  | 33 | Intercept | -347,011 | 535,288 | -,648 | 0,519 |
|  |  |  |  |  | Qmx5-10 | -1,020 | ,418 | -2,439 | 0,018 |
|  |  |  |  |  | T5-10 | 42,587 | 40,542 | 1,050 | 0,298 |
|  |  |  |  |  | DQmx | 1,132 | ,687 | 1,647 | 0,105 |
|  |  |  |  |  | 01 Stadl-St.Georgen | -65,992 | 124,687 | -,529 | 0,599 |
|  |  |  |  |  | 02 St.Georgen - Murau | -161,399 | 126,404 | -1,277 | 0,207 |
|  |  |  |  |  | 03 Murau-Unzmarkt | -19,902 | 122,012 | -,163 | 0,871 |
|  |  |  |  |  | 04 Unzmarkt-Judenburg | -31,345 | 119,724 | -,262 | 0,794 |
|  |  |  |  |  | 05 RW Fisching | 599,694 | 141,400 | 4,241 | 0,000 |
|  |  |  |  |  | 06 Fisching-Pregbach | 6,050 | 127,850 | ,047 | 0,962 |
|  |  |  |  |  | 07 Pregbach-Leoben | 0^a^ |  |  |  |
|  |  |  |  | 34 | Intercept | -341,875 | 535,567 | -,638 | 0,526 |
|  |  |  |  |  | T5-10 | 42,307 | 40,557 | 1,043 | 0,301 |
|  |  |  |  |  | DQmx | 1,116 | ,685 | 1,630 | 0,109 |
|  |  |  |  |  | Qmx4-10 | -1,013 | ,418 | -2,427 | 0,018 |
|  |  |  |  |  | 01 Stadl-St.Georgen | -66,061 | 124,759 | -,530 | 0,599 |
|  |  |  |  |  | 02 St.Georgen - Murau | -161,410 | 126,479 | -1,276 | 0,207 |
|  |  |  |  |  | 03 Murau-Unzmarkt | -20,046 | 122,093 | -,164 | 0,870 |
|  |  |  |  |  | 04 Unzmarkt-Judenburg | -31,573 | 119,803 | -,264 | 0,793 |
|  |  |  |  |  | 05 RW Fisching | 599,516 | 141,472 | 4,238 | 0,000 |
|  |  |  |  |  | 06 Fisching-Pregbach | 5,987 | 127,920 | ,047 | 0,963 |
|  |  |  |  |  | 07 Pregbach-Leoben | 0^a^ |  |  |  |
| electrofishing | YOY density autumn | all | 31 | 35 | Intercept | 351,325 | 105,105 | 3,343 | 0,003 |
|  |  |  |  |  | Qmx4-10 | -,947 | ,279 | -3,389 | 0,003 |
|  |  |  |  |  | 01 Stadl-St.Georgen | -52,614 | 101,800 | -,517 | 0,610 |
|  |  |  |  |  | 02 St.Georgen - Murau | -114,643 | 106,286 | -1,079 | 0,292 |
|  |  |  |  |  | 03 Murau-Unzmarkt | -27,537 | 101,811 | -,270 | 0,789 |
|  |  |  |  |  | 04 Unzmarkt-Judenburg | 33,989 | 96,030 | ,354 | 0,727 |
|  |  |  |  |  | 05 RW Fisching | 482,481 | 113,624 | 4,246 | 0,000 |
|  |  |  |  |  | 06 Fisching-Pregbach | 48,125 | 106,286 | ,453 | 0,655 |
|  |  |  |  |  | 07 Pregbach-Leoben | 0^a^ |  |  |  |
|  |  |  |  | 36 | Intercept | 349,234 | 104,713 | 3,335 | 0,003 |
|  |  |  |  |  | Qmx5-10 | -,942 | ,278 | -3,389 | 0,003 |
|  |  |  |  |  | 01 Stadl-St.Georgen | -52,339 | 101,807 | -,514 | 0,612 |
|  |  |  |  |  | 02 St.Georgen - Murau | -114,406 | 106,288 | -1,076 | 0,293 |
|  |  |  |  |  | 03 Murau-Unzmarkt | -27,057 | 101,805 | -,266 | 0,793 |
|  |  |  |  |  | 04 Unzmarkt-Judenburg | 34,530 | 96,032 | ,360 | 0,722 |
|  |  |  |  |  | 05 RW Fisching | 482,481 | 113,627 | 4,246 | 0,000 |
|  |  |  |  |  | 06 Fisching-Pregbach | 48,362 | 106,288 | ,455 | 0,653 |
|  |  |  |  |  | 07 Pregbach-Leoben | 0^a^ |  |  |  |

Table S6_1 Model results of the final models: sample number (N), coefficient estimates, standard error (SE), T values and significance (p values). Model number (Nr.) refers to the models in the main text. ^a^ redundant (set to zero)

Table S6_2 Results of paired samples comparison for total and YOY densities (Wilcoxon rank test); Bold values indicate the site with the higher density (p < 0.1)

**Supporting Information Data S7**

**Reported effects of flushing operations on fish**

Table S7_1 shows a compilation of different flushing events. Trends in fish densities are given irrespectively of the size of the effect. The compilation shows that ecological optimised sediment management often has no negative effect on fish. Oxygen depletion and very high SSC are always accompanied by a reduction in fish densities.

| **Reservoir** | **River** | **Distance downstream (km)** | **Reference** | **Duration** | **SSC_max_** | **O_2_** | **Trend in fish densities** | **Con-trolled** |
| --- | --- | --- | --- | --- | --- | --- | --- | --- |
| Cancano^1^ | Adda River 2010 S1/II | 7/14.2 | Brignoli et al. 2015, Espa et al 2016 | 46 days | 30.2 g.L^-1^ |  | Spring: +  Autumn: - | Yes |
| Cancano^1^ | Adda River 2011 S1/II | 7/14.2 | Brignoli et al. 2015, Espa et al 2016 | 53 days | 68.9 g.L^-1^ |  | Spring: -  Autumn: - | Yes |
| Cancano^1^ | Adda River 2010 S5/V | 24/22.9 | Brignoli et al. 2015, Espa et al 2016 | 46 days | 3.3 g.L^-1^ |  | Spring: -  Autumn: + | Yes |
| Cancano^1^ | Adda River 2011 S5/V | 24/22.9 | Brignoli et al. 2015, Espa et al 2016 | 53 days | 6.2 g.L^-1^ |  | Spring: +  Autumn: - | Yes |
| Cancano^1^ | Adda River 2012 | 24/22.9 | Espa et al 2016 | 40 days | 38.2 g.L^-1^ |  | Spring: -  Autumn: + | Yes |
| Cancano^1^ | Adda River 2010 S5/VI | 24/28.2 | Brignoli et al. 2015 | 46 days | 3.3 g.L^-1^ |  | Spring: -  Autumn: + | Yes |
| Cancano^1^ | Adda River 2011 S5/VI | 24/28.2 | Brignoli et al. 2015 | 53 days | 6.2 g.L^-1^ |  | Spring: n.a.  Autumn: - | Yes |
| Cancano^1^ | Adda River 2010 S5/VIII | 24/46.7 | Brignoli et al. 2015 | 46 days | 3.3 g.L^-1^ |  | Spring: =  Autumn: + | Yes |
| Cancano^1^ | Adda River 2011 S5/VIII | 24/46.7 | Brignoli et al. 2015 | 53 days | 6.2 g.L^-1^ |  | Spring: -  Autumn: + | Yes |
| Valgrosina^2^ | Roasco Creek 2006 | 5,3/5,3 | Crosa et al. 2010, Espa et al. 2013 | 12-13 days | > 50 g.L^-1^ | >10 mg.L^-1^ | - | Yes |
| Valgrosina^2^ | Roasco Creek 2007 | 5,3/5,3 | Crosa et al. 2010, Espa et al. 2013 | 12-13 days | 10-15 g.L^-1^ | >10 mg.L^-1^ | - | Yes |
| Valgrosina^2^ | Roasco Creek 2006 | 5,3/6,0 | Crosa et al. 2010, Espa et al. 2013 | 12-13 days | > 50 g.L^-1^ | >10 mg.L^-1^ | - | Yes |
| Valgrosina^2^ | Roasco Creek 2007 | 5,3/6,0 | Crosa et al. 2010, Espa et al. 2013 | 12-13 days | 10-15 g.L^-1^ | >10 mg.L^-1^ | - | Yes |
| Valgrosina^2^ | Roasco Creek 2008 | 5,3/5,3 | Espa et al. 2013 | 12-13 days | 10-20 g.L^-1^ |  | - | Yes |
| Valgrosina^2^ | Roasco Creek 2008 | 5,3/6,0 | Espa et al. 2013 | 12-13 days | 10-20 g.L^-1^ |  | + | Yes |
| Valgrosina^2^ | Roasco Creek 2009 | 5,3/5,3 | Espa et al. 2013 | 12-13 days | 40-50 g.L^-1^ |  | + | Yes |
| Valgrosina^2^ | Roasco Creek 2009 | 5,3/6,0 | Espa et al. 2013 | 12-13 days | 40-50 g.L^-1^ |  | - | Yes |
| Upper Yarra^3^ | Yarra River 1991 S3 | 0,25 | Doeg und Koehn 1994 | 7 months | 4.61 g.L^-1^ | Oxygen depletion likely | - | No |
| Upper Yarra^3^ | Yarra River 1991 S5 | 2,1 | Doeg und Koehn 1994 | 7 months | 0.20 g.L^-1^ | Oxygen depletion likely | - | No |
| Upper Yarra^3^ | Yarra River 1991 S6 | 2,7 | Doeg und Koehn 1994 | 7 months | 0.16 g.L^-1^ | Oxygen depletion likely | - | No |
| Sernio^4^ | Adda 2009 | 5.0 | Espa et al. 2012, 2015 | 16 days | 4.8 g.L^-1^ |  | BT Spring: -  BH Spring: + | Yes |
| Sernio^4^ | Adda 2010 | 5.0 | Espa et al.2012, 2015 | 6 days | 2.7 g.L^-1^ |  | BT Autumn: +  BH Autumn: + | Yes |
| Fionnay | Danse de Bagnes 1991 |  | Gester & Rey 1994 |  | 150 ml.L^-1^ | No oxygen depletion | - |  |
| Livigno | Spöl 1990 |  | Gester & Rey 1994 |  | 15 ml.L^-1^ | No oxygen depletion | = |  |
| Palagnedra | Melezza 1991a |  | Gester & Rey 1994 |  | 900 ml.L^-1^ | < 1 % | - |  |
| Palagnedra | Melezza 1991 b |  | Gester & Rey 1994 |  | 150 ml.L^-1^ | No oxygen depletion | = |  |
| Verbois | Rhone 2012 |  | Grimardias et al. 2012, 2017 | 12 days | ca. 50 g.L^-1^ | > 7 mg.L^-1^ | - |  |
| Spencer | Niobrara River 1994 |  | Gutzmer et al. 1996 |  | 20 mg.L^-1^ |  | - | Yes |
| Spencer | Niobrara River 1995 |  | Gutzmer et al. 1996 |  | < 10 mg.L^-1^ |  | + | Yes |
| Spencer^5^ | Niobrara River 1993-2001 |  | Gutzmer et al. 2002 |  | n.a. |  | - | Yes |
| Spencer | Niobrara River |  | Hesse und Newcombe 1982 |  | 21.8 g.L^-1^ | < 4 mg.L^-1^ | - | No |
| Margaritze | Möll 1960 | 0.5 - 23 | Liepolt 1961 |  | 3.6 – 66.2 g.L^-1^ |  | = | No |
| Elandsdrift |  |  | Palmer & O'Keefe 1990 |  | 2.8g.L^-1^ |  | - |  |
| Speicher Paal | Paalbach 2013 | 0 | Parthl et al. 2013 | 12 days | 17.4 g.L^-1^ | >8.3 mg.L^-1^ | + | Yes |
| Speicher Paal | Paalbach 2013 | 5.3 | Parthl et al. 2013 | 12 days | 8.7 g.L^-1^ | >9.1 mg.L^-1^ | + | Yes |
| Urstein | Salzach 1999 | 0.5 km | Petz-Glechner et al. 2003 | 1 day | 13.2 g.L^-1^ |  | + | Yes |
| Urstein | Salzach 1999 | 8.6 km | Petz-Glechner et al. 2003 | 1 day | 13.2 g.L^-1^ |  | - | Yes |
| Urstein | Salzach 1999 | 9.7 km | Petz-Glechner et al. 2003 | 1 day | 13.2 g.L^-1^ |  | + | Yes |
| Urstein | Salzach 1999 | 12.1 km | Petz-Glechner et al. 2003 | 1 day | 13.2 g.L^-1^ |  | - | Yes |
| Urstein | Salzach 1999 | 15.3 km | Petz-Glechner et al. 2003 | 1 day | 13.2 g.L^-1^ |  | + | Yes |
| Madesimo^6^ | Scalcoggia Stream | Diluting flows | Quadroni et al.2016 | 3 days |  |  | BT -  BH - |  |
| Madesimo | Scalcoggia Stream | 5.1 | Quadroni et al.2016 | 3 days | 17 g.L^-1^ | >8.0 mg.L^-1^ | BT -  BH + |  |
| Margaritze | Möll 1965 |  | Sampl 1999 |  | “very high” |  | - | No |
| Bolgenach | Bregenzer-ach1995 |  | Schotzko 2012 |  | 1.000 ml.L^-1^ |  | - |  |
| Vermunt | Ill | 0.5 | Schotzko 2012 |  | 75 g.L^-1^ |  | = |  |
| Vermunt | Ill – Residual reach | 1.5 | Schotzko 2012 |  | 50 g.L^-1^ |  | = |  |
| Margaritze | Möll 1995 |  | Seifert 1995 | 3 days | 300 g.L^-1^ |  | - | No |

Table S7_1 Reported effects of flushing operations; SSC_max_ – maximal reported SSC; *long term investigation – no negative impact of ecological optimised sediment management on fish;

BT – brown trout, BH - bullhead

^1^ SSC was measured at stations S1 (7 km below the dam) and S5 (24 km below the dam), fish densities were measured at stations II, V, VI, and VIII; comparisons were made between spring and autumnal fish samples before and after a flushing event; data may be biased by fishing/restocking

^2^ SSC and O_2_ were measured 5.3 km below the dam; seasonal effects on fish densities are likely (before samples were taken in the beginning auf August, after samples were taken mid/end September)

^3^ SSC are mean values; no specific pre-release data had been collected; before data were from opportunistic studies six and three years before the flushing event

^4^SSC was measured 0.9 km below dam; 2009 before-samples for fish were taken at a different site 2 km upstream; comparisons were made between spring and autumnal fish samples before and after a flushing event;

^5^ long term investigation – the species composition has not dramatically changed, with the possible exception of decrease of river shiners

^6^ not effected by SSC, only affected by diluting flows

Brignoli, M., Espa, P., Quadroni, S., Torretta, V., & Ionescu, G. (2015). Environmental impact of reservoir desilting operation. UPB Sci. Bull. Ser. D Mech. Eng., 77(2), 257-270.

Crosa, G., Castelli, E., Gentili, G., & Espa, P. (2010). Effects of suspended sediments from reservoir flushing on fish and macroinvertebrates in an alpine stream. Aquatic Sciences, 72(1), 85.

Doeg, T. J., & Koehn, J. D. (1994). Effects of draining and desilting a small weir on downstream fish and macroinvertebrates. River Research and Applications, 9(4), 263-277.

Espa, P., Brignoli, M. L., Crosa, G., Gentili, G., & Quadroni, S. (2016). Controlled sediment flushing at the Cancano Reservoir (Italian Alps): management of the operation and downstream environmental impact. Journal of environmental management, 182, 1-12.

Espa, P., Brignoli, M. L., Prato, A. P., Crosa, G., Quadroni, S., & Gentile, G. (2012) Field Investigation of controlled sediment flushing at Sernio pondage. 9th ISE 2012,Vienna

Espa, P., Castelli, E., Crosa, G., & Gentili, G. (2013). Environmental effects of storage preservation practices: controlled flushing of fine sediment from a small hydropower reservoir. Environmental management, 52(1), 261-276.

Espa, P., Crosa, G., Gentili, G., Quadroni, S., & Petts, G. (2015). Downstream ecological impacts of controlled sediment flushing in an Alpine valley river: a case study. River research and applications, 31(8), 931-942.

Gerster S. & P. Rey (1994) Ökologische Folgen von Stauraumspülungen. BUWAL Schriftenreihe Umwelt 219. 49 pp.

Grimardias, D., Guillard, J., & Cattanéo, F. (2017). Drawdown flushing of a hydroelectric reservoir on the Rhône River: Impacts on the fish community and implications for the sediment management. Journal of Environmental Management, 197, 239-249.

Grimardias, D., Guillard, J., De Bono, C. N., Diouf, S., Zanasco, J. L., & Cattanéo, F. Impact of the Verbois reservoir flushing in (2012): how did fish survive 'the apocalypse'?.

Gutzmer, M. P., King, J. W., & Overhue, D. P. (1996). Environmental impacts in the vicinity of Spencer Hydropower Dam during sluicing activities in the Niobrara River, Nebraska.

Gutzmer, M. P., King, J. W., Overhue, D. P., &Chrisp, E. Y. (2002). Fish species-richness trends in the Niobrara River, Nebraska, below the Spencer dam.

Hesse, L. W., & Newcomb, B. A. (1982). Effects of flushing Spencer Hydro on water quality, fish, and insect fauna in the Niobrara River, Nebraska. North American journal of fisheries management, 2(1), 45-52.

Liepolt R. (1961) Biologische Auswirkung der Entschlammung eines Hochgebirgsstausees in einem alpinen Fließgewässer. Wasser und Abwasser 110-133

Palmer R. & J.A.Y. O'Keefe (1990). Transported material in a small river with multiple impoundments. Freshwater biology, 24(3), 563-575.

Parthl et al. (2013) Fischökologisches Monitoring Paalbach

Petz-Glechner R., Petz W., Kainz E. & O. Lapuch (2003) Die Auswirkung von Stauraumspülungen auf Fische. Natur in Tirol 12, 74-93

Quadroni, S., Brignoli, M. L., Crosa, G., Gentili, G., Salmaso, F., Zaccara, S., &Espa, P. (2016). Effects of sediment flushing from a small Alpine reservoir on downstream aquatic fauna. Ecohydrology, 9(7), 1276-1288.

Sampl H. (1999) Kärntner Umweltbericht 1999. Amt der Kärntner Landesregierung. 553 pp

Schotzko N. (2012) Entleerung und Entlandung von Stauräumen: Herausforderungen und Lösungsansätze für ein gewässerverträgliches Feststoffmanagement anhand von Fallbeispielen aus Vorarlberg. Sachverständigentagung des Österreichischen Fischereiverbandes

Seifert K. (1995) Die Auswirkungen der Spülung des Margaritzenspeichers auf den aquatischen Lebensraum und den Fischbestand der Möll. 24 pp
